# A New Source of Diterpene Lactones From *Andrographis paniculata* (Burm. f.) Nees—Two Endophytic Fungi of *Colletotrichum* sp. With Antibacterial and Antioxidant Activities

**DOI:** 10.3389/fmicb.2022.819770

**Published:** 2022-02-28

**Authors:** Na Li, Dan Xu, Rui-Hua Huang, Jian-Yun Zheng, You-Yan Liu, Bin-Sheng Hu, Yuan-Qin Gu, Qin Du

**Affiliations:** Medicinal Plant Biotechnology Laboratory, College of Chinese Medicine, Guangzhou University of Chinese Medicine, Guangzhou, China

**Keywords:** *Andrographis paniculata* (Burm. f.) Nees, endophytic fungi, *Colletotrichum* sp., antibacterial activity, antioxidant activity, diterpene lactone

## Abstract

Endophytic fungi of medicinal plants are abundant, and their metabolites often have antioxidant, antibacterial, and antitumor effects and can produce secondary metabolites identical or similar to those of their hosts, which can mitigate the problem of insufficient supply of medicinal plants. In this study, we screened endophytic fungi for strains that produce the same diterpene lactones as *Andrographis paniculata* based on their biological activity. Firstly, the dominant group of endophytic fungi of *Andrographis paniculata* was screened and pathogenicity was studied using Koch’s rule. Secondly, DPPH, ABTS, OH, PTIO radical scavenging, and FRAP assays were used to detect the antioxidant activity of the extracellular extracts of the strains, and total phenol and total flavonoid contents of the strains with high antioxidant capacity were determined. *S. aureus*, *B. subtilis*, *E. coli*, and *P. aeruginosa* were used to determine the antibacterial activity of the mycelial extracts of the strains. Finally, the secondary metabolites of the mycelial extracts of the strains were examined by high-performance liquid chromatography. The results showed that 32 strains of *Andrographis paniculata* were relatively isolated > 70% and non-pathogenic. Extracellular extracts of strains AP-1 and AP-4 showed vigorous antioxidant activity, and AP-4, AP-12, AP-47, and AP-48 showed antibacterial activity against four strains of bacteria. The HPLC results indicated that the mycelial extracts of AP-4 and AP-12 contained diterpene lactones. The two endophytic fungi were recognized as *Colletotrichum* sp. The study successfully obtained diterpene lactones from the endophytic fungus of *Andrographis paniculata* and confirmed the feasibility of using endophytic fungal strains to produce active substances consistent with the host. It was also useful for exploring endophytic fungi and medicinal plants. The relationship provides theoretical guidance.

## Introduction

**FIGURE 1:**
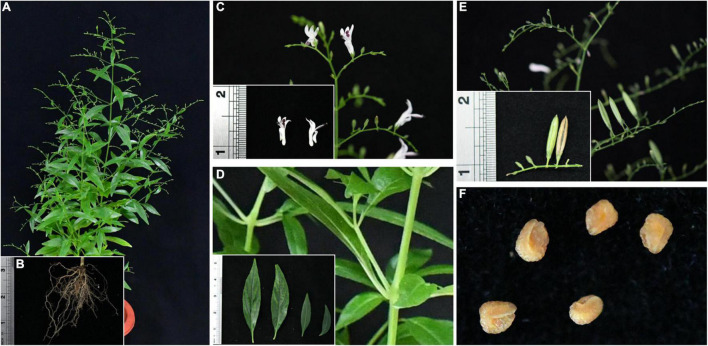
An annual herb of Traditional Chinese medicine for clearing heat and detoxifying. **(A)** AP: plant, **(B)** roots, **(C)** flowers, **(D)** stems and leaves, **(E)** mature and immature fruits, and **(F)** seeds.

*Andrographis paniculata* (Burm. f.) Nees (AP) (Figure 1) is a herbaceous medicinal plant in the family Acaciaceae, native to India and Sri Lanka (Kaushik et al., 2020; Kumar et al., 2021). Now, it is widespread in the tropical countries of Southeast Asia, including India, Thailand, Myanmar, Indonesia, and southern China (Kandanur et al., 2019). AP is utilized in various traditional oriental medicines and is known as “Kalmegh” in India, “Fah Tha Lai” in Thailand, and “Hempedabumi” in Malaysia (Murthy and Dalawai, 2021). The dried aboveground part of AP, known as “Chuan-Xin-Lian” in the 2020 edition of the Chinese Pharmacopoeia, is a traditional Chinese herb (Chinese Pharmacopoeia Commission, 2020). AP is known as the “king of bitterness,” with the effect of clearing heat and detoxifying, cooling the blood, and reducing swelling (Yang et al., 2020). It is traditionally used to treat snake bites and poisonous stings of insects, common cold, fever, gastrointestinal disorders, liver disorders, and upper respiratory tract infections (Handa et al., 1986; Jarukamjorn and Nemoto, 2008; Jiao et al., 2019). With the advancement of modern pharmacological research, AP has been shown to have antimalarial (Banerjee et al., 2020; Dwivedi et al., 2021), antibacterial (Dai et al., 2019), anti-inflammatory (Sun et al., 2019), antioxidant (Mussard et al., 2019), anti-HIV (Uttekar et al., 2012), hypotensive (Yang et al., 2010), and hypoglycemic (Jaiyesimi et al., 2020) effects. At the time of COVID-19 in China, the National Health Commission has published six consecutive editions of the “Pneumonia Treatment Protocol for Novel Coronavirus Infection (Trial),” of which the sixth edition recommends the use of “Xi-Yan-Ping” injection (the raw material is AP lactone total sulfonate) for the treatment of clinically severe and critical cases (Nan et al., 2020). Researchers from diverse countries started to study the inhibitory effect of AP on COVID-19 extensively.

AP contains numerous bioactive compounds such as diterpene lactones, flavonoids, and polyphenols, among which diterpene lactones and flavonoids are high in content, especially diterpene lactones, which are the most representative medicinal components (Murthy and Dalawai, 2021). The 2020 version of the Chinese Pharmacopoeia specifies the total lactone content consisting of andrographolide (AD), neandrographolide (NAD), 14-deoxyandrographolide (DAD), and 14-deoxy-11,12-didehydroandrograph (DDAD) as the index for evaluating the quality standard of AP; qualified dried samples of AP herb should have a total lactone content of 1.5% (Chinese Pharmacopoeia Commission, 2020). The research of flavonoids mainly focuses on *in vitro* antioxidation (Wu et al., 2008). Because of its rich medicinal activity, the commercial value of AP in China is exceptionally lofty. However, single genetic background, narrow geographic distribution, and destruction of wild germplasm habitats have limited the germplasm diversity of AP, coupled with the degradation of farm varieties by self-crossing, which has seriously hampered the large-scale cultivation, resulting in short supply of AP (Chen et al., 2014). In addition, the problem of continuous crop barriers in the cultivation and production of AP has seriously affected its yield and quality (Hong et al., 2021). In order to save land resources, we need to find a culture method that can replace artificial cultivation to produce valuable medicinal components.

Endophyte fungi originally referred to fungi that colonize the interior of host plants without causing overt symptoms. The discovery of an endophytic fungus named Andre Paclitaxel in 1993 is considered a landmark achievement in the field of endophytic bacteria in research (Gómez and Luiz, 2018), which directly proves the connection between planting endophytic fungi and plant physiological activity process. Being dependent on the symbiosis theory, endophytic fungi and host plants have adapted to each other and influenced each other during the long evolutionary process. This symbiosis provides a variety of biosynthetic pathways for endophytic fungi, resulting in rich structural types of secondary metabolites of plant endophytic fungi. At present, A variety of structural types such as alkaloids, terpenoids, sterols, anthraquinones, flavonoids, phenolics, perylene derivatives, furandiones and cyclic peptides have been found to be synthesized. These ingredients will also have corresponding beneficial biological functions and applications, such as antibacterial, antiviral, anticancer, antioxidant, insecticidal, antidiabetic, and immunosuppressive effects (Deshmukh et al., 2015; Gao et al., 2018). Especially in antioxidation and antibacterial aspects, it has always been a hot spot in the research direction of activity. Li et al. (2017) isolated five new polyhydroxy hydroanthraquinone derivatives from the marine endophytic fungus Talaromyces. Wang et al. (2019) studied the extracellular polysaccharides of Alternaria tenuissima from *Angelica sinensis*; they found that it is composed of d-galacturonic acid, rhamnose, d-mannose, glucose, and d-galactose in a ratio of 0.45:3.02:3.25:1.0:0.95. Tianpanich et al. (2011) isolated isocoumarin and phthalate from the endophytic fungus *Colletotrichum sp.* The above compounds have been found to have intense free radical scavenging activity. In recent years, more and more compounds with antimicrobial activity have been discovered, such as peptides (Tejesvi et al., 2016), anthraquinones, and terpenes (Radiæ and Strukelj, 2012). Undoubtedly, the active metabolites of plant endophytic fungi are potential precursors of natural antioxidant and antimicrobial drugs with important research significance and economic value.

Endophytic fungi may acquire certain corresponding genes of the host by ingesting the host DNA, which gives them the ability to produce the same metabolic components as the host plant (Wani et al., 2015), and also act as initiators to rapidly activate specific genes in the secondary metabolic pathways of medicinal plants to accumulate large amounts of active ingredients (Zhai et al., 2017). Studies have shown that sustainable production of metabolites such as Taxol (Zhou et al., 2010), vincristine, vinblastine, camptothecin, and podophyllotoxin (Mohana Kumara et al., 2012) for axenic fermentation of endophytic fungi has been reported. In recent years, researchers have identified gentiopicrin-producing endophytic fungi from *Gentiana macrophylla* (Yin et al., 2009) and tanshinone and tanshinolenic acid-producing endophytes from *Salvia miltiorrhiza* (Shi et al., 2019); fermentation using endophytes from *Huperzia* plants was obtained, producing huperzine (Le et al., 2020), and paeoniflorin and chlorogenic acid were successively extracted from *Paeonia lactiflora* as well as from endophytic fungi of *Eucommia ulmoides* Oliver (Chen et al., 2010; Cheng et al., 2018). Endophyte production active ingredients provide an alternative method that can narrow the large number of processes associated with plant harvesting to meet the growing demand for compounds (Zaki et al., 2019; Sang et al., 2020). In this sense, the use of endophytic fungi to produce the same medicinal ingredients as the host can circumvent the risk of long growth time and low yield of the plant itself, as well as reduce the complex genetic pathways than *in vitro* tissue organ and cell culture, and can provide an environmentally friendly, relatively simple, and inexpensive alternative route.

AP is a tropical medicine with high economic value, and endophytic fungi are one of its essential components. In this study, the endophytic fungi of AP planted in the experimental field were isolated many times, and the dominant species were selected. After the experimental removal of pathogenic fungi causing the apparent disease in the host plant, the remaining 32 strains were fermented and cultured for preliminary extraction of secondary metabolites. The extracellular extracts (EXEs) were assayed for antioxidant activity and flavonoid and phenolic content. The antibacterial activity of the mycelial extracts (MEs) of strains was assayed. The potential of the crude mycelium extract for the production of AP diterpene lactones was evaluated by HPLC. Our research can provide a new way to obtain the medicinal ingredients of AP. It is the first step toward the industrial extraction and production of the antioxidant and antibacterial active ingredients of the endophytic fungus of AP and the research and development of drugs.

## Materials and Methods

### Collection of Plant Materials

Healthy samples of AP were collected from the “Yao-Wang-Shang” experimental field (23°5′87.726″N, 113°40′42.723″E) of Guangzhou University of Chinese Medicine, Guangzhou City, Guangdong Province, China, in October 2019. Thirty plants with the highest active ingredient content were randomly selected at the beginning of the flowering period. AP (Murthy and Dalawai, 2021) were uprooted and stored in a refrigerator at 4°C in the Pharmaceutical Plant Biotechnology Laboratory and processed as the sample within 24 h.

### Isolation of Endophytic Fungi

The surface of the leaves was disinfected, soaked first in 75% ethanol for 30 s and then placed in 4% sodium hypochlorite solution for 15–20 min with shaking, followed by washing with sterile water three times. The leaves were cut into square blocks of about 5 mm × 5 mm and inoculated on a potato dextrose agar (PDA) medium. Finally, the plates were incubated in a constant temperature incubator at 28°C for 8–10 days. The openings of the blank PDA medium were placed on an ultra-clean table as blank control. The final rinsing solution was applied on the medium as rinsing control. The tissue blocks at the end of the detoxification were placed on the culture for 20 min and then removed as the blot control. If no mycelium growth was observed in the three controls, it was ensured that the isolated fungus was an endophytic fungus of AP. After mycelium growth appeared on the edge of the tissue block, different mycelium forms were picked and cultured on the new PDA medium until a single form of the strain was obtained. This operation is replicated 10 times, and fungus No. 1 is named “AP-1,” and so on. For each strain, four parts of the mycelium were taken, three parts were stored in 30% glycerol at −4°C, and one part was inoculated in a slant medium for preservation.

### Screening of Dominant Groups and Detection of Endophytic Fungal Pathogenic Strain

The endophytic fungi preserved on the slant medium were cultured on fresh PDA medium at 28°C for 6–10 days. The morphology, color, texture, and other characteristics of the colonies were observed during incubation, and the mycelium was stained with lactophenol cotton blue orchid and observed under an Olympus CX23 light microscope (Olympus Chin Co., Ltd., Beijing) for preliminary identification and classification of the strains according to the Manual of Fungal Identification (Wei, 1979). Relative frequency (RF) analysis was used to determine the dominant taxa of endophytic fungi at each site: RF (%) = number of an endophytic fungal strain in the sample/total number of isolated strains × 100, and endophytic fungi with RF > 70% were selected to continue the next experiment.

The sterilized leaves were then cut into two equal parts and placed in Petri dishes with sterile filter paper moistened with sterile water, one of which was inoculated with the dominant endophytic fungus at the leaf cut, and the other was not inoculated as a control. The leaves were incubated in a constant temperature incubator at 28°C for 5–7 days; if the leaves showed decay, whitening, and mycelial growth, the strain was initially judged to be pathogenic. The Koch rule (Walker et al., 2006) was then used to verify that the strain was the pathogenic fungus and to discard these strains.

### Fermentation of Endophytic Fungal and Extraction of Crude Product

The preserved strains were activated and cultured, and 3 endophytic fungal pieces were punched with a punch with a diameter of 6 mm and put into a 500-ml Erlenmeyer flask containing 400 ml of sterilized potato dextrose broth (PDB) solution and fermented at 28°C, pH 7.0, and 150 r/min. Each strain was fermented repeatedly for three times, according to the DNS assay of reducing sugar, until the fermentation is completed. Endophytic fungal fermentation liquid was vacuum filtered and divided into two parts: mycelial medium and fermentation culture medium. Ethyl acetate (1:1, w/w) was added to the fermentation culture medium for 3 extractions; the ethyl acetate layers were combined and spin dried on a rotary evaporator at 45°C, 100 r/min; and an appropriate amount of pure methanol (MeOH) was added to dissolve the crude product to make 20 mg/ml EXEs. The mycelia were dried in an oven at 45°C, ground with liquid nitrogen, and passed through a No. 4 sieve, 65 meshes; 2.000 ± 0.005 g powder was accurately weighed; and 40 ml of 80% MeOH was added, allowed to stand for 30 min, and then syndicated 3 times, 30 min each time, and let to stand at room temperature and then filtered with suction. The filtrate was spin dried on a rotary evaporator at 45°C and 100 r/min. An appropriate amount of pure MeOH was added to dissolve the crude product to prepare 20 mg/ml of MEs. The EXEs and MES were stored in a refrigerator at 4°C.

### Determination of Antioxidant Activity of Extracellular Extracts

The antioxidant activity of EXEs was evaluated by the 2,2-diphenyl-1-picrylhydrazyl (DPPH), 2,2-azino-bis(3-ethylbenzothiazoline-6-sulfonic acid (ABTS), hydroxyl radical (OH), 2-phenyl-4,4,5,5-tetramethylimidazoline-1-oxyl 3-oxide radical (PTIO), and ferric reducing antioxidant power (FRAP) methods, which were improved according to Li (2017), Biondi et al. (2018), and Gulcin (2020). The EXE samples with high antioxidant data were selected, gradient diluted, and analyzed for IC_50_ values (the concentration at which 50% scavenging of DPPH, ABTS, hydroxyl, PTIO, and ferric was achieved) to evaluate the antioxidant activity of EXEs. The positive control is ascorbic acid (V*c*), and 3 parallel experiments were repeated for each EXE sample. Ferrous sulfate was purchased from the National Institute for Food and Drug Control (NIFDC, Beijing, China).

#### 2,2-Diphenyl-1-Picrylhydrazyl Radical Scavenging Activity

A DPPH test solution was prepared, and 2 ml EXEs (20 mg/ml) was added to the test tube containing 2 ml DPPH (0.2 mM) solution. The solution was incubated in the dark for 30 min at room temperature, and the absorbance was measured at 517 nm with a MAPADA UV-1800 ultraviolet spectrophotometer (MAPADA Instruments Co., Ltd., Shanghai, China) and recorded as A_1_. 2 ml of the DPPH test solution was mixed with 2 ml of MeOH, and the absorbance was recorded as A_0_. 2 ml of MeOH was mixed with 2 ml of the EXEs, and the absorbance was recorded as A_2_. The calculation formula of the scavenging rate (S%) is S (%) = 1-(A_1_-A_2_)/Ao × 100%.

#### 3-Ethylbenzothiazoline-6-Sulfonic Acid Radical Scavenging Activity

0.2 ml ABTS (7.4 mM) solution was mixed with 0.2 ml potassium persulfate solution (2.6 mM), stood for 12 h in the dark, and diluted with MeOH to an absorbance value of 0.7 ± 0.02 at 734 nm, which is the ABTS test solution. 0.4 ml of the sample was taken and mixed with 4 ml ABTS test solution, light was avoided for 20 min at room temperature, and the absorbance value measured was recorded at a wavelength of 734 nm as A_1_. 0.4 ml of MeOH was mixed with 4 ml of the ABTS test solution, and the measured absorbance value is A_2_. The calculation formula of the scavenging rate is as follows: S (%) = 1-A_1_/A_2_ × 100%.

### Hydroxyl Radical Scavenging Activity

The amount of each solution added is shown in Table 1. The solution was incubated at 37°C for 15 min, and the absorbance A_0_ was measured at 510 nm. The absorbance A_1_ and A_2_ was measured as shown in Table 1; the calculation formula for the scavenging rate (S%) is S (%) = 1-(A_1_-A_2_)/A_0_ × 100%.

**TABLE 1:** Hydroxyl radical scavenging reagent addition table.

	A_0_ (ml)	A_1_ (ml)	A_2_ (ml)
FeSO_4_	0.4	0.4	0.4
Acid-ethanol	0.4	0.4	0.4
EXEs		0.2	0.2
Deionized water	4.8	4.6	4.6
H_2_0_2_	0.4	0.4	

### 2-Phenyl-4,4,5,5-Tetramethylimidazoline-1-Oxyl 3-Oxide Radical Scavenging Activity

A total of 800 μl of PTIO (0.15 mg/ml) test solution was mixed with 200 μl of MeOH to measure the A_0_ value at 557 nm. A total of 800 μl of the PTIO test solution was taken and mixed with 200 μl of EXEs, and gradient dilution was performed according to the fading condition. The sample concentration was diluted stepwise according to the fading situation. If the color cannot be faded immediately, a 37°C water bath is required for 2 h. After the mixed solution fades, the absorbance A_1_ is measured. The calculation formula of the scavenging rate (S%) is as follows: S (%) = A0-A_1_/A_0_ × 100%.

#### Ferric Reducing Antioxidant Power

Acetic acid buffer (pH 3.6), Fe^3+^-tripyridinetriazine (TPTZ) solution (10.0 mM), and FeCl_3_ solution (20.0 mM) were mixed as FRAP test solutions in the ratio of 10:1:1. 5 μl of ferrous sulfate with concentrations of 0.2, 0.4, 0.6, 0.8, 1.0, and 1.2 mM was added to a 96-well plate, and 180 μl of the FRAP test solution was added. Moreover, the absorbance value A was measured at 593 nm by holding at 37°C for 10 min. The linear regression curve is obtained from the absorbance (y) and the concentration of ferrous sulfate (x). The results were expressed as FeSO_4_^⋅^7H_2_O equivalents/ml per ml of different strains of EXEs, abbreviated as mmol Fe^2+^/ml.

### Determination of Total Phenols and Total Flavonoids of Extracellular Extracts

The total phenol content was determined by the Folin–Ciocalteu colorimetric method with a slight modification. Using gallic acid (NIFDC, Beijing, China) solution as the standard, a Folin phenol reagent and 5% Na_2_CO_3_ solution were added, mixed well, and placed in a water bath at 30°C for 30 min, and the absorbance was measured at 745 nm. The equation of the standard curve was obtained using the mass concentration (x) and absorbance (y) of gallic acid in the concentration range of 0.00–0.10 mg/ml. A total of 75 μl of LEXEs was added to 75 μl of Folin and 3.6 ml of 5% sodium carbonate solution, mixed well, and bathed in water at 30°C for 30 min, and the absorbance was measured at 745 nm. The total phenol content is expressed in milligrams of gallic acid per milliliter of culture solution, that is, μg GAE/ml.

The NaOH-Al(NO_3_)_3_-NaNO_2_ colorimetric method was utilized to detect the content of flavonoids. 0.5 ml of the sample (20 mg/ml) was mixed with 5 ml of MeOH, after which 0.5 ml NaNO_2_ (0.5 mM) and 0.5 ml Al(NO_3_)_3_ (0.3 mM) solution were added to the mixture. After 6 min, 0.5 ml NaOH solution (1.0 M) was added to the mixture and cultured for 30 min at room temperature. Rutin (NIFDC, Beijing, China) was used as a standard, and the absorbance was measured at a wavelength of 500 nm. The equation of the standard curve was obtained using the mass concentration (x) and absorbance (y) of rutin in the concentration range of 0.00–0.20 mg/ml. The results are expressed in rutin equivalents (RE), which is μg RE/ml.

### Detection of Antibacterial Activity of Mycelial Extracts

The indicator pathogens *S. aureus* (ATCC 6538) and *B. subtilis* (ATCC 6633) which are Gram-positive bacteria and *E. coli* (ATCC 8739) and *P. aeruginosa* (ATCC 9027) which are Gram-negative bacteria were purchased from Guangdong Microbial Culture Collection Center, activated, and cultured on Luria-Bertani (LB) medium.

#### Determination of *in vitro* Antibacterial Assays

The antibacterial activity of MEs was evaluated using the Oxford cup method, as described by Zhang et al. (2021). The indicator bacteria were used to prepare a bacterial suspension of 1 × 10^5^ CFU/ml for later use. A total of 100 μl of bacterial solution was spread evenly on the LB medium, four Oxford cups were placed in each petri dish, and 100 μl of MEs was added and incubated in a 37°C incubator for 24 h. A vernier caliper “cross method” was used to measure the zone of inhibition. Penicillin-streptomycin was set as the positive control, MeOH as the solvent control; the thrice-repeated experiments were done for each indicator pathogen for each sample; and the diameter of the inhibition zone (excluding the diameter of the Oxford cup) was used to evaluate the antibacterial ability of MEs. This was repeated for each of the tests in triplicate.

#### Determination of Minimum Inhibitory Concentration and Minimum Bactericidal Concentration

The minimum inhibitory concentration (MIC) of MEs was assessed by the 96-well plate method (Wiegand et al., 2008; Dydak et al., 2021). 1 × 10^5^ CFU/ml indicator solutions of 100 μl were added to the 96-well enzyme labeling plate in sequence, 100 μl of MEs filtered with a 0.22-μm microporous membrane was added to the first well (20 mg/ml), 100 μl was aspirated to the second well after blowing well, and so on to the ninth well, and 100 μl of the mixture was aspirated and discarded. Then, ME was diluted into nine concentrations, 20, 10, 5, 2.5, 1.25, 0.625, 0.312, 0.156, and 0.0781 mg/ml. The LB liquid medium with bacterial suspension was used as positive control (PC), and the liquid medium containing only LB as negative control (NC), and incubated in the incubator at 37°C for 24 h; the inhibition effect was judged by visual observation of the clarity of the bacterial solution: a cloudy bacterial solution is judged to have bacterial growth, and the dividing concentration between clarity and cloudiness is MIC (Wiegand et al., 2008). A total of 50 μl of the culture solution was aspirated in the clarification well and spread on a LB culture plate, and incubated in the incubator at 37°C for 24 h. The minimum dilution concentration of the fermentation solution with no bacterial growth is the minimum bactericidal concentration (MBC); three groups were set for each parallel test solution.

### Detection of Diterpene Lactones in Mycelial Extracts by HPLC

#### Preparation of *Andrographis paniculata* (Burm. f.) Nees Plant Samples, Andrographolide Standard, and Mycelial Extract Samples

Referring to the 2020 edition of the Chinese Pharmacopoeia (Chinese Pharmacopoeia Commission, 2020), the aboveground parts of AP samples were collected at the beginning of flowering, baked at 45°C in an oven until constant weight, powdered, and sieved using a No. 4 sieve; about 0.500 ± 0.002 g of powder was soaked for 1 h with 40% MeOH in 25 ml, weighed and treated with an ultrasound (power 250 w, frequency 33 kHz) for 30 min, let to cool, weighed again, and filtered with 40% MeOH. The weight loss was made up of 40% MeOH, and the filtrate was filtered through a 0.22-μm microporous membrane. The standard substance of AD (NIFDC, Beijing, China) substance was accurately measured, and MeOH was added to make a solution containing 0.3002 mg AD per 1 ml and passed through a 0.22-μm microporous membrane. MEs passed through a 0.22-μm microporous filter membrane to obtain the sample tested by HPLC.

#### Determination of Andrographolide, Neandrographolide, 14-Deoxyandrographolide, and 14-Deoxy-11,12-Didehydroandrograph Content

The identification and concentration of AD, NAD, DAD, and DDAD in crude bioactive fractions were determined by HPLC (Shimadzu China Co., Ltd., Beijing) using a Hydrosphere C18 column (250 mm × 4.6 mm, 5 μm, Shimadzu China Co., Ltd., Beijing) equipped with a dual-wavelength detector and Workstation software. About 5 μl of the sample of defined concentration was injected into the HPLC column and gradient elution using acetonitrile (A)–pure water (B): (0–15 min, 20–25% A; 15–30 min, 25–28% A; 30–60 min, 28–40% A; 60–65 min, 40–85% A), with a column oven temperature of 30°C. The wavelength length is 205 nm, and the flow velocity is 1 mg/ml. The data of the peak area concentration of the standard AD obtained were used to estimate the quantity of AD in MEs, according to the regulations of the Chinese Pharmacopoeia of the 2020 edition (Chinese Pharmacopoeia Commission, 2020), with AD as the internal reference, and the relative retention time of NAD, DAD, and DDAD is calculated. The retention time should be within 5% of the specified value and meet the above requirements. The peak area of AD can be used as a control, and the correction factor (Table 2) can be multiplied to calculate the content of AD, NAD, DAD, and DDAD in accordance with the pharmacopeia, and the content is expressed in mg of active ingredients per g of the dry weight of mycelium, abbreviated as mg/g DW.

**TABLE 2:** Relative retention time and correction factor of AP diterpene lactone HPLC.

Ingredients to be tested	Relative retention time	Correction factor
AD	1.00	1.00
NAD	1.95	1.12
DAD	2.18	0.79
DDAD	2.25	0.63

### Molecular Identification of Strongly Active Endophytic Fungi

The fungal DNA was extracted using Solarbio^®^ Fungal Genomic DNA Extraction Kit D2300 (Soleibao Biotechnology Co., Ltd., Guangzhou), and qualified DNA was extracted for PCR amplification of rDNA-ITS. The PCR reaction system is 50 μl, including primer ITS1 (5′-TCCGTAGGTGAACCTGCGG-3′) and ITS4 (5′-TCCTCCGCTTATTGATATGC-3′) 1 μl each, 2 × Taq PCR mix (including blue dye) 25 μl, genomic DNA 1 μl, and ddH_2_O 20 μl. The reaction conditions were as follows: pre-denaturation at 94°C for 5 min, denaturation at 94°C for 30 min, annealing at 55°C for 30 s, extension at 72°C for 45 s, 35 cycles, final extension at 72°C for 10 min, and storage at 4°C. After 1% agarose gel electrophoresis detection, the qualified PCR products were sent to Shenggong Bioengineering Co., Ltd. (Shanghai), for sequencing. Then, the products were spliced, and the optimized ITS sequence was submitted to the NCBI website, using the BLAST program for comparison.

### Statistical Analysis of the Data

All data in the chart are expressed as the mean ± SD of three independent replicate data samples, and the standard error is calculated using SPSS 20.0 software (IBM, Armonk, NY, United States). Analysis of variance (ANOVA) was performed, and the statistical difference between the treatments was analyzed by Duncan’s multiple-range test (*p* < 0.05). IC_50_ determination was carried out using GraphPad Prism 8.0 software. Hierarchical cluster analysis (HCA) was realized using the MATLAB^®^ numerical software. The heat map is produced by the online mapping website Bioinfo Intelligent Cloud^1^.

## Results

### Screening of Endophytic Fungi Strains

**FIGURE 2:**
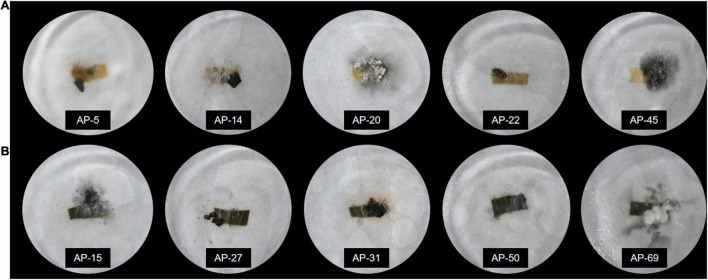
Pathogenicity detection of endophytic fungi after inoculation with endophytic fungi. **(A)** The leaves show yellowing, whitening, rotting, and other diseases. **(B)** The leaves remained fresh and unchanged after inoculation with endophytic fungi.

A total of 252 endophytic fungal strains were isolated from 10 experiments on AP leaves, and the relative frequency (RF) (%) analysis of the strains revealed that 74 endophytic fungal strains with IR > 70% were classified as the dominant strains of AP. The pathogenicity of the dominant strains was verified, and 42 strains were found to cause disease phenomena such as decay and whitening of the host (Figure 2). After eliminating the pathogenic fungi, follow-up experiments on the remaining 32 dominant endophytic fungi continued to be conducted. After preliminary identification, 32 strains from ***Colletotrichum*** sp., ***Hypoxylon*** sp., ***Nodulisporium*** sp., ***Diaporthe*** sp., and ***Peroneutypa*** sp. were identified.

### Antioxidant Activity of Extracellular Extracts

**FIGURE 3:**
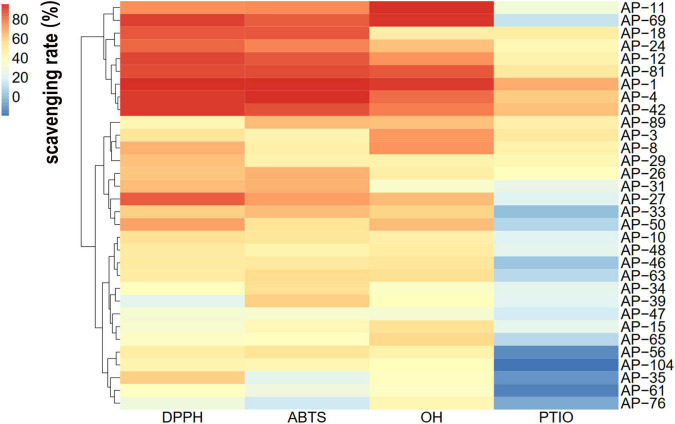
Hierarchical cluster analysis (HCA) of the antioxidant activity of extracellular extracts (EXEs) was obtained from 32 fungal endophytes.

In the current study, the antioxidant activity of 32 dominant endophytic EXEs was evaluated using the DPPH, ABTH, OH, and PTIO radical scavenging methods. The overall antioxidant effects of 32 dominant endophytic fungi were analyzed by HCA to statistically identify groups with similar performance. The results are shown in Figure 3. As can be seen, the strains of AP-1, AP-4, AP-11, AP-12, AP-18, AP-24, AP-42, AP-69, and AP-81 were clustered under the same group and have the most potent antioxidant activity.

**FIGURE 4:**
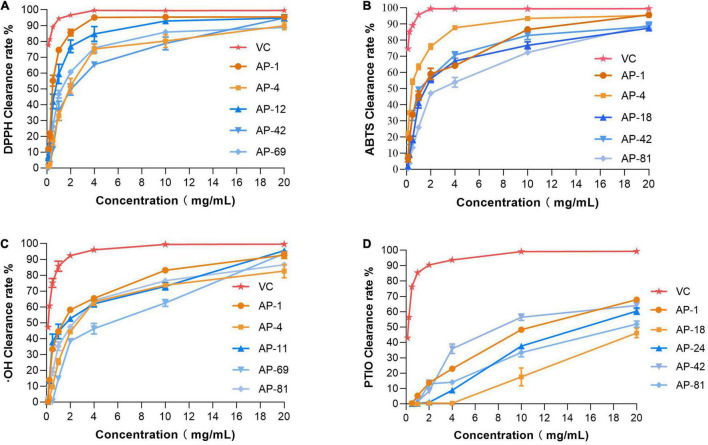
Antioxidant activities of different concentrations of EXEs. **(A)** DPPH radical scavenging activity. **(B)** ABTS radical scavenging activity. **(C)** Hydroxyl radical scavenging activity. **(D)** PTIO radical scavenging activity.

The EXEs of the five strains with the strongest radical scavenging ability of DPPH, ABTS, OH, and PTIO were selected for further antioxidant activity tests. EXEs were diluted gradually, and their IC_50_ value was determined and compared. As shown in Figures 4A–D, the radical scavenging ability of EXEs decreases as the concentration decreases. In the DPPH, ABTS, and OH radical scavenging experimental groups, when the concentration of the test sample is 20 mg/ml, the clearance rate of EXEs and V*c* of the strain is not much different. When the concentration is gradually reduced, the gap between the clearance rates of EXEs and V*c* has also increased significantly (Figures 4A–C). In the PTIO radical scavenging experimental group, the gap is more prominent.

**FIGURE 5:**
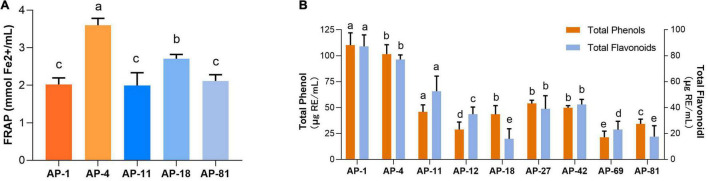
The content of FRAP, total phenols, and total flavonoids of EXEs. **(A)** FRAP of EXEs. **(B)** The content of total phenols and total flavonoids of EXEs.

In addition, the IC50 measurements of EXEs for the five strains with the highest ability to scavenge DPPH, ABTS, OH, and PTIO radicals are summarized, as shown in Table 3. In the DPPH group, AP-1 had the smallest IC_50_ value of 0.468 ± 0.051 mg/ml (*p* < 0.05). In the ABTS group, the IC_50_ values of AP-4 and AP-42 were the same, while the data were significantly lower compared to the others (*p* < 0.05) at 0.324 ± 0.012 and 0.274 ± 0.105 mg/ml, respectively. In the OH group, the IC_50_ value of AP-1 was 1.429 ± 0.292, which was the smallest value among the five strains (*p* < 0.05). In the PTIO group, the strain with the smallest IC_50_ was AP-42 (3.67 ± 0.249 mg/ml). In the concentration range of 0.00–1.00 mg/l, the standard equation of FeSO_4_-7H_2_O *y* = 0.3835x + 0.0262, *R*^2^ = 0.9996, was obtained, and the activity of FRAP was measured. The results of EXEs of the five strains with the strongest scavenging ability are shown in Figure 5A. The FRAP of AP-42 was significantly higher than those of other strains at 3.600 ± 0.181 mmol Fe^2+^/ml (*p* < 0.05). In conclusion, three strains of AP, AP-1, AP-4, and AP-42, had potent antioxidant activity.

**TABLE 3:** IC_50_ value from four antioxidant activity assays DPPH, ABTS, OH, and PTIO from the EXEs.

Strains	IC_50_ value			
	DPPH	ABTS	OH	PTIO
AP-1	0.468 ± 0.051^c^	1.933 ± 0.375^b^	1.429 ± 0.292^d^	11.817 ± 0.063^a^
AP-4	1.408 ± 0.172^a^	0.324 ± 0.012^d^	1.779 ± 0.129^c^	–
AP-11	–	–	1.141 ± 0.046^b^	–
AP-12	0.572 ± 0.027^b^	1.087 ± 0.196^c^	–	–
AP-18	–	–	–	11.218 ± 0.415^a^
AP-24	–	–	–	9.720 ± 0.897^c^
AP-42	1.591 ± 0.411^a^	0.274 ± 0.105^d^	–	3.670 ± 0.249^d^
AP-69	0.816 ± 0.057^b^	–	4.256 ± 0.673^a^	
AP-81	–	3.187 ± 0.634^a^	1.759 ± 0.040^c^	10.824 ± 0.061^b^
V*c*	0.030 ± 0.030^d^	0.032 ± 0.001^e^	0.032 ± 0.003^e^	0.039 ± 0.002^e^

*“–“ means not detected (n = 3). “–” indicates that the content is not detected. The same column with different lowercase letters was significantly different by Duncan’s multiple-range test (p < 0.05).*

### Total Phenol and Total Flavonoid Content of Extracellular Extracts

The standard equation of gallic acid *y* = 0.2229x–0.1717, *R*^2^ = 0.9992, was measured in the concentration range of 0–0.1 mg/ml. The standard equation of rutin y = 12.488x + 0.0105, *R*^2^ = 0.9997, was obtained in the concentration range of 0–0.2 mg/ml. The standard curves of gallic acid and rutin showed a good linear relationship. Using the standard equation, the total phenol and total flavonoid contents of the eight strains of EXEs with antioxidant activity in the first echelon (AP-1, AP-4, AP-11, AP-12, AP-24, AP-42, AP-69, AP-81) were obtained (Figure 5B). Among them, the EXE total phenol content of AP-1 and AP-4 is 110.194 ± 11.800 and 101.576 ± 9.073 μg GAE/ml, and the total flavonoid content is 87.217 ± 8.854 and 77.101 ± 3.510 μg RE/ml, respectively, both of which are significantly higher compared with other strains (*p* < 0.05).

### Antioxidant Activity of Extracellular Extracts

**FIGURE 6:**
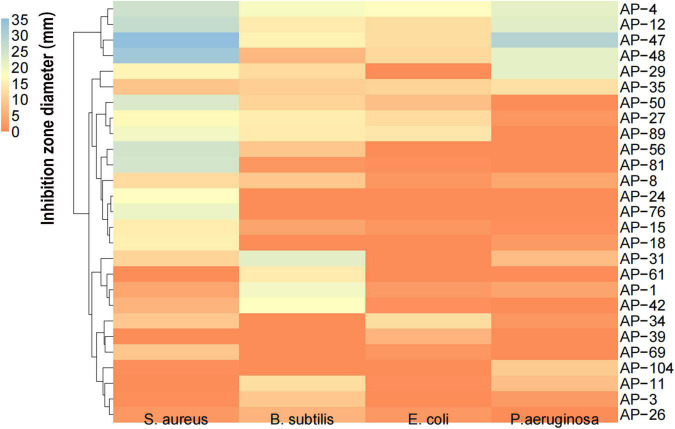
HCA of antioxidant capacity of 32 dominant endophytic fungi mycelial extractions (MEs) against *S. aureus*, and *B. subtilis*, *E. coli*, and *P. aeruginosa*.

**FIGURE 7:**
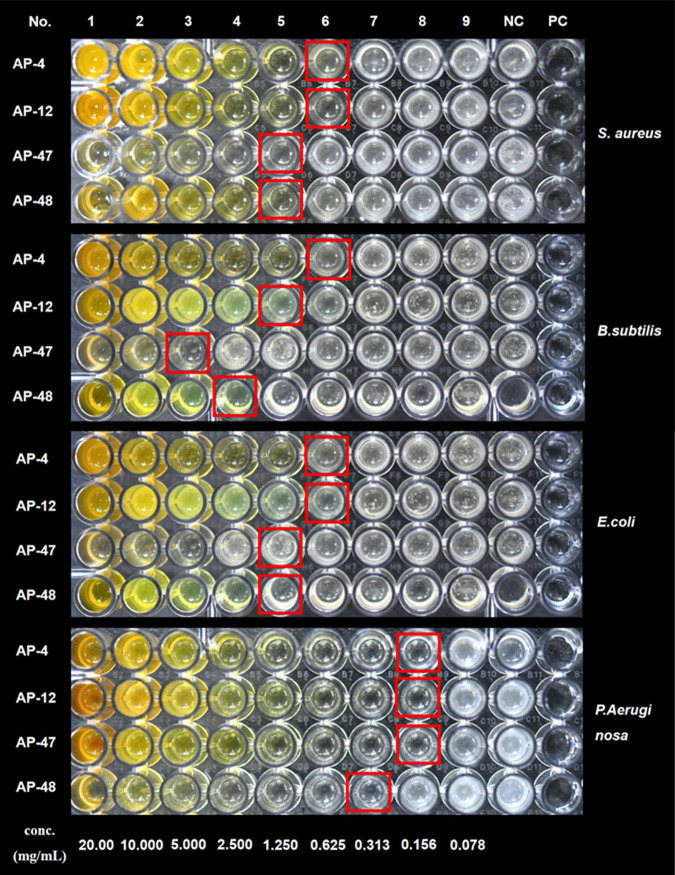
MIC of AP-4, AP-12, AP-47, and AP-48 against *S. aureus*, and *B. subtilis*, *E. coli*, and *P. aeruginosa*.

The size of the inhibition zone of *S. aureus*, *B. subtilis*, *E. coli*, and *P. aeruginosa* of the MEs of 32 dominant endophytic fungi was determined by the Oxford cup method. HCA was carried out on the antibacterial effects of 32 dominant endophytic fungi; the results are presented in Figure 6. The results showed that the MEs of the four fungi AP-4, AP-12, AP-47, and AP-48 have strong antibacterial abilities. The MIC of their MEs was further determined; the results are shown in Figure 7, and the data are summarized in Table 4. It can be seen that the antibacterial efficacy of the four strains against *S. aureus*, *B. subtilis*, *E. coli*, and *P. aeruginosa* is AP-4 > AP-12 > AP-48 > AP-47. Among them, AP-4 is effective for *E. coli*, *P. aeruginosa*, and *S. aureus*, it has an excellent inhibitory effect, and its MIC value is 0.625 mg/ml. The bacterium liquid in the three wells before the MIC concentration is placed on fresh LB medium, and the MBC value is determined after 24 h of culture. It is shown in Table 4 that the bactericidal effect of AP-4 and AP-12 is stronger than that of AP-47 and AP-48. Experiments have proved that the two strains AP-4 and AP-12 have potent antibacterial activity, and the possibility of producing AP diterpene lactones is high, and the test can be continued.

**TABLE 4:** Determination of minimum inhibitory concentration (MIC) and minimum bactericidal concentration (MBC) of AP-4, AP-12, AP-47, and AP-48 (*n* = 3).

	*S. aureus*		*B. subtilis*		*E. coli*		*P. aeruginosa*	
	MIC (mg/ml)	MBC (mg/ml)	MIC (mg/ml)	MBC (mg/ml)	MIC (mg/ml)	MBC (mg/ml)	MIC (mg/ml)	MBC (mg/ml)
AP-4	0.625	2.500	0.625	2.500	0.625	1.250	0.156	1.250
AP-12	0.625	2.500	1.250	5.000	0.625	2.500	0.156	0.313
AP-47	1.250	5.000	5.000	10.000	1.250	10.00	0.156	0.313
AP-48	1.250	2.500	2.500	5.000	1.250	1.250	0.313	2.500

### Detection of Diterpene Lactones in Mycelial Extracts

**FIGURE 8:**
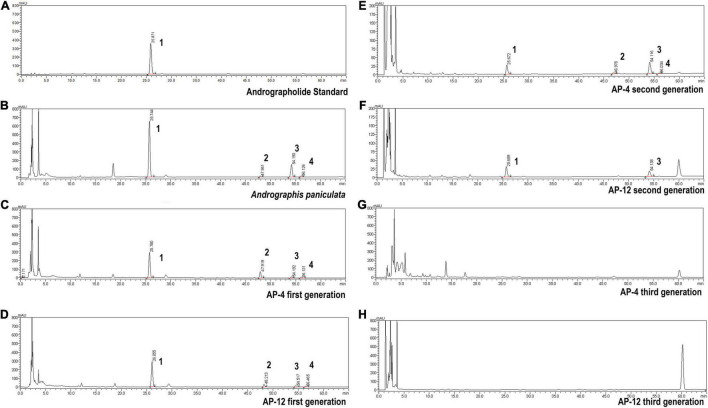
High-performance liquid chromatograms of AD (1), NAD (2), DAD (3), and DDAD (4). **(A)** Andrographolide standard. **(B)** *Andrographis paniculata.* **(C)** AP-4 first generation. **(D)** AP-12 first generation. **(E)** AP-4 second generation. **(F)** AP-12 second generation. **(G)** AP-4 third generation. **(H)** AP-12 third generation.

**FIGURE 9:**
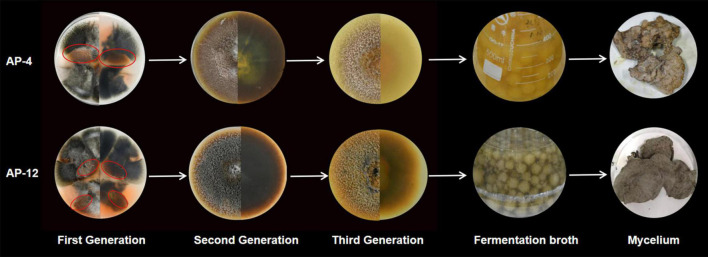
The strain morphology, fermentation morphology, and mycelium morphology of AP-4 and AP-12.

According to the testing requirements of the 2020 edition of *the Chinese Pharmacopoeia*, the contents of AD, NAD, DAD, and DDAD in AD standards and samples were determined, and the high-performance liquid chromatograms are as shown in Figure 8. The peak areas of AD standards were used as an internal reference; the values were multiplied by the corresponding correction factors (Table 2). The relative contents of AD, NAD, DAD, and DDAD could be calculated; the results are shown in Table 5. It is shown in Figure 9 that the retention time of MEs was the same as that of the standard and AP extract. MEs of both AP-4 and AP-12 first-generation strains contained AD, NAD, DAD, and DDAD with the highest AD content of 30.089 ± 0.992 and 28.617 ± 0.641 mg/g DW (Table 5). The NAD content of the first-generation ME of strain AP-4 was about 7 times higher than that of the AP plant at 7.585 ± 0.512 mg/g DW, while the NAD content of the first-generation strain of AP-12 was 3 times higher than that of AP (3.277 ± 0.111 mg/g DW). With successive generations of the strain, the diterpene lactone component contained in it was substantially decreased. After the first purification, the NAD content was undetectable in the ME of AP-4, while both NAD and DDAD contents were undetectable in the ME of AP-12. It is worth noting that although the strains of the first generation contained considerable diterpene lactones, the strains selected for the first generation in this study were the first isolated from AP leaves (Figure 9) and had not been purified, so the extraction of active ingredients by endophytic fungi would inevitably be affected by contamination and degeneration.

**TABLE 5:** The content of andrographolide (AD), neandrographolide (NAD), 14-deoxyandrographolide (DAD), and 14-deoxy-11,12-didehydroandrograph (DDAD) (*n* = 3).

	Content (mg/g DW)			
	AD	NAD	DAD	DDAD
AP	63.798 ± 1.440^a^	1.086 ± 0.032^c^	13.878 ± 0.232^a^	0.827 ± 0.048^a^
AP-4 first generation	30.089 ± 0.992^b^	7.585 ± 0.512^a^	1.414 ± 0.042^c^	0.690 ± 0.069^b^
AP-12 first generation	28.617 ± 0.641^b^	3.277 ± 0.111^b^	1.479 ± 0.082^c^	0.686 ± 0.067^b^
AP-4 second generation	3.018 ± 0.168^d^	0.512 ± 0.008^d^	3.577 ± 0.064^b^	–
AP-12 second generation	3.460 ± 0.149^d^	–	1.697 ± 0.086^c^	–

*“–” indicates that the content is not detected. The same column with different lowercase letters was significantly different by Duncan’s multiple-range test (p < 0.05).*

### Molecular Identification of Strongly Active Endophytic Fungi

**FIGURE 10:**
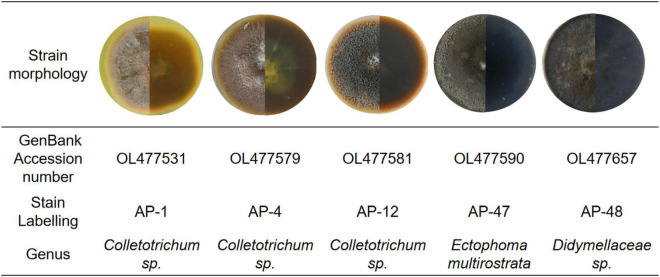
Basic information of AP-1, AP-4, AP-12, AP-47, and AP-48 and their first-generation mycelial morphology.

In this study, two strains AP-1 and AP-4 with strong antioxidant activity and high total phenol and total flavonoid content were obtained. Among the four fungi with potent antibacterial activity—AP-4, AP-12, AP-47, and AP-48—AP-4 and AP-12 can produce AP diterpene lactones. After amplification and sequencing, the specific information of each active strain was obtained. AP-1, AP-4, and AP-12 belonged to *Colletotrichum*, AP-47 belonged to *Ectophoma multirostrata*, and AP-48 corresponded to *Didymellaceae.* The obtained sequences were uploaded to GenBank, and the accession numbers and related information are shown in Figure 10.

## Discussion

For decades, the active ingredients in herbal medicine AP have been discovered and developed into various anti-pathogenic drugs and formulations. However, the single germplasm of Chinese AP and the restriction of crop rotation barriers make it difficult to guarantee the quality of the host plant (Yang et al., 2020). People realize that it is sometimes difficult to meet the demand just to develop functional ingredients from medicinal plants. Therefore, finding a broader source of biologically active metabolites is a hot spot for researchers (Zhai et al., 2017). Endophytic fungi are widely found in medicinal plants and are undoubtedly a vast treasure trove of natural active ingredients (Jia et al., 2016). However, the variety and number of endophytic fungi present throughout the growth cycle of medicinal plants are enormous. Finding strains capable of producing host medicinal components is a daunting project. Therefore, endophytic fungi should be screened to some extent. Our ultimate goal is to obtain strains with the same medicinal composition as the host, requiring the target strains to be easy to isolate and culture. In this study, we used the most basic PDA medium to cultivate endophytic fungi. After repeating the culture 10 times, a total of 252 endophytic fungi were isolated. According to the plant–microbe symbiosis system, endophytic fungi interact with each other during long-term coevolution with the host (Wani et al., 2015), then the selected endophytic fungi should coexist with the host for a long time and have a clear growth advantage. We determined the relative isolation frequency (RF) of 252 endophytic fungi and selected dominant endophytic fungal communities with RF > 70%. Dominant endophytic fungal communities colonize heavily during the life cycle of the host and are relatively unaffected by environmental changes (Knapp et al., 2019). Jia (2014) calculated that the structure of the endophytic fungal flora of *Coix lacryma-jobi* was significantly correlated with the active ingredients of the host plant, triglyceride, and coixol, by establishing a correlation analysis and linear regression analysis model, proving that the dominant flora was a close connection with the active ingredients of the host. This result can confirm that it is meaningful to prioritize the selection of dominant fungi in this study and can prevent the interference of miscellaneous fungi. Endophytic fungi are a type of fungi that exist in plants and do not cause obvious diseases to plants (Mohana Kumara et al., 2012). According to this theory, we inoculated sterile AP leaves with 74 dominant endophytic fungi and used Koch’s rule to remove pathogenic risk strains from the dominant endophytic fungi to finally obtain 32 non-pathogenic dominant endophytic fungi (Figure 2). After a series of screenings, we narrowed the scope of the endophytic fungi to be studied and set the stage for the in-depth study of biological activity.

As a fungus capable of producing the same active ingredients as the host, it should have the same activity as the host (Kouipou Toghueo and Boyom, 2019). AP is rich in flavonoids and diterpene lactones, and the target endophytic fungi should have explicit antioxidant and antibacterial activities. It is well known that excessive oxygen free radicals (reactive oxygen species and reactive nitrogen species) can cause damage to biomolecules in cells, making antioxidants a hot topic of research (Valko et al., 2006; Del Río, 2015). New antioxidant assays are constantly being developed. As research progressed, it was found that the traditional DPPH and ABTS radicals are both nitrogen-centered groups. Therefore, they are more suitable for the “reactive nitrogen species scavenging” model rather than the “reactive oxygen species scavenging” model (Li, 2017). The PTIO radical scavenging experiment is a novel method for detecting oxygen free radicals. It has four major advantages such as oxygen-centered radicals, physiological aqueous solution, and simple and direct measurement with low interference of the test sample (Li, 2017). In this study, the antioxidant activity of 32 endophytic fungal EXEs was comprehensively evaluated by using PTIO and OH assays as indicators for scavenging reactive nitrogen, DPPH and ABTS assays as indicators for scavenging reactive oxygen species, and FRAP assay as an indicator for the ability to reduce “Fe^3+^.” The antioxidant activity of EXEs from eight endophytic fungal strains, AP-1, AP-4, AP-11, AP-12, AP-24, AP-42, AP-69, and AP-81, was evaluated (Figure 3). According to known research results, the content of total phenols and total flavonoids is highly correlated with antioxidant activity (Zhang et al., 2020). After testing the total phenols and total flavonoids of the EXEs of 8 strains of endophytic fungi, AP-1 and AP-4 contained relatively high total phenols and total flavonoids (Figure 5B). These findings indicate that the endophytic fungi AP-1 and AP-4 derived from AP have the potential to develop new antioxidant drugs. Of course, further research is recommended to purify and characterize the structure of biologically active ingredients. One of the major characteristics of Andrographis diterpene lactones is that they are insoluble in water, so we tested the antibacterial ability of endophytic fungal MEs. The antibacterial activity of 32 endophytic fungal MEs was evaluated using *S. aureus*, *B. subtilis*, *E. coli*, and *P. aeruginosa*, which are commonly used in antibacterial assays of AP, as indicator bacteria. The results showed that AP-4, AP-12, AP-47, and AP-48 had potent antibacterial activity (Figure 6), which can be utilized as diterpene lactone assay strains for subsequent experiments.

Since the isolation of a paclitaxel-producing endophytic fungus from *Taxus brevifolia* by Stierle et al. (1993) in the United States, there has been a desire to screen for bioactive substances similar to their hosts from endophytic fungi of some economically important medicinal plants. Therefore, in order to alleviate the shortage and oversupply of some medicinal plants, researchers have increased the development and application of endophytic fungal resources. After decades of development, Ming et al. (2012) isolation of a *Trichoderma* endophytic fungus from the root of the medicinal herb *Salvia miltiorrhiza* is reported for the first time. The fermentation products of this fungus were confirmed to contain tanshinone I and tanshinone IIA by HPLC and LC-HRMS/MS in comparison with authentic standards. Ancheeva et al. (2020) review provides an overview of secondary metabolites from endophytic fungi with pronounced biological activities covering the literature between 2010 and 2017. Those rich and diverse compounds were discovered from the fermentation products of endophytic fungi, and the identification of the configuration used HPLC, LC-MS or GC-MS, and other detection methods. As for AP, there is no report on its endophytic fungus and active product research. In this study, the fermentation products of four endophytic fungi with strong antibacterial activity were tested by HPLC with reference to the assay method used in the above literature, and two strains AP-4 and AP-12 with Andrographis diterpene lactone-producing components were successfully identified, with Andrographis lactone content up to 30.089 ± 0.992 and 28.617 ± 0.641 mg/g DW (Table 5). This undoubtedly once again verified the feasibility of using endophytic fungi to obtain host-related active ingredients. Through molecular identification, the two endophytic fungi belong to *Colletotrichum* sp. When the species is further compared, it is found that neither of the two endophytic fungi can achieve a 100% match with the existing sequences in the database, so they could only be tentatively named by genus. *Colletotrichum* is a fungus that is widespread in plants and can cause plant anthracnose, and it is a phytopathogenic fungus (Kim and Shim, 2019). However, by observation, it did not cause anthracnose, at least in appearance of AP, and did not show obvious signs of disease. These changes may be due to a mechanism of the long-term synergistic symbiosis between *Colletotrichum* and AP, the exact cause of which needs to be further investigated. It is worth noting that although AP-4 and AP-12 can produce considerable AP diterpene lactones, the strains undergoing fermentation culture are all isolated strains of the first generation and have not been purified. Therefore, it is more susceptible to contamination by miscellaneous fungi during fermentation. When the second-generation strains were HPLC tested, the content of diterpene lactones was significantly reduced. The fungus of *Colletotrichum* was highly susceptible to degeneration, and the fungus lost the ability to produce diterpene lactones after three generations. It can be inferred that AP-4 and AP-12 do not synthesize diterpene lactones *per se* but that direct transmission of genes involved in the secondary metabolite synthesis pathway occurs during symbiosis with AP. This transmission may occur during “symbiotic organism–host” or “parasitic organism–host” interactions (Zeng et al., 2000). The “symbiosis theory” also suggests that once a biochemical pathway used in secondary metabolism is exhibited in a symbiont, it can be used by other organisms to exhibit interactions and “co-evolution” (Faeth and Sullivan, 2003). The host can transmit its genetic material or biochemical information to the endophytic fungus so that it has to some extent the same or similar metabolic pathways as the host and so that it produces certain specific substances. The mechanism has yet to be further studied and needs to be confirmed by definite evidence. For the degradation of strains, this can be resolved by changing the culture method of the strain and mutagenesis. The addition of host cells as additives in the fermentation process of endophytic fungi, coculture with strains, or the use of fungi as inducers to grow in synergy with host plants can, to some extent, result in higher yields of secondary metabolites. Mutagenesis of endophytic fungi using heavy ion beam, ultraviolet light, and chemical mutagens is an emerging method for industrial strain transformation in recent years (Zou et al., 2015), and the use of mutagenesis to generate mutations in strains can solve the problem of strain degradation and obtain stable and high-yielding industrial strains.

## Conclusion

In this study, endophytic fungi of *Andrographis paniculata* were isolated for the first time, through a series of bioactivity-related assays; two strains, AP-1 and AP-4, with significant antioxidant activity and high total phenol and total flavonoid yields were obtained. Four strains with potent antibacterial activity were obtained: AP-4, AP-12, AP-47, and AP-48; two strains AP-4 and AP-12 from *Colletotrichum* containing diterpene lactones were screened. This is a preliminary report on the production of diterpene lactones by endophytic fungi from AP, which has important pioneering significance for the industrial extraction and production of active ingredients of AP and drug development. However, the degradation problem of the two diterpene lactone-producing strains of *Colletotrichum* sp. was also found to seriously hinder the synthesis of active substances. Therefore, this study suggests the possibility of further research to mutagenize the two strains using chemical and physical means of production so that they can remain fresh and minimize the negative effects of strain passage.

## Data Availability Statement

The datasets presented in this study can be found in online repositories. The names of the repository/repositories and accession number(s) can be found in the article/Supplementary Material.

## Author Contributions

NL, DX, R-HH, and QD conceived and designed the experiments. DX and Y-YL screened 32 dominant endophytic fungi. NL and R-HH completed the antioxidant and antibacterial experiments. J-YZ and B-SH cultivated AP for experimentation. NL and Y-QG analyzed the data. NL and DX wrote the draft manuscript. QD provided financial support for the experiments and directed the writing. All authors contributed to the article and approved the submitted version.

## Conflict of Interest

The authors declare that the research was conducted in the absence of any commercial or financial relationships that could be construed as a potential conflict of interest.

## Publisher’s Note

All claims expressed in this article are solely those of the authors and do not necessarily represent those of their affiliated organizations, or those of the publisher, the editors and the reviewers. Any product that may be evaluated in this article, or claim that may be made by its manufacturer, is not guaranteed or endorsed by the publisher.

## Footnotes

1
http://www.ehbio.com/Cloud_Platform/front/#/
