# Supplementary material for: A New Source of Diterpene Lactones From Andrographis paniculata (Burm. f.) Nees—Two Endophytic Fungi of Colletotrichum sp. With Antibacterial and Antioxidant Activities
Source: Front Microbiol. 2022 Feb 28;13:819770. doi: 10.3389/fmicb.2022.819770 (PMC8918950; doi:10.3389/fmicb.2022.819770)

Supplementary Material

1. **Supplementary Tables**

# Supplementary Table 1 The scavenging rate of DPPH, ABTS, OH, and PTIO radicals of 32 dominant endophytic fungi (n=3).

| Strains | scavenging rate (%) | | | |
| --- | --- | --- | --- | --- |
|  | DPPH | ABTS | OH | PTIO |
| AP-1 | 95.647±0.969 | 95.647±0.969 | 92.747±2.072 | 68.270±1.767 |
| AP-3 | 53.837±2.520 | 44.203±2.041 | 73.223±2.309 | 51.235±1.187 |
| AP-4 | 92.49±1.825 | 95.247±1.085 | 82.65±4.218 | 61.466±1.769 |
| AP-8 | 67.217±1.366 | 46.17±1.090 | 74.367±1.208 | 45.415±2.986 |
| AP-10 | 54.57±3.142 | 52.797±1.397 | 46.923±1.606 | 20.321±1.989 |
| AP-11 | 75.88±1.443 | 76.887±1.904 | 95.537±0.957 | 29.471±2.260 |
| AP-12 | 90.797±2.580 | 87.423±3.610 | 74.113±1.567 | 45.040±2.650 |
| AP-15 | 32.81±4.772 | 42.223±4.471 | 54.857±1.826 | 23.088±1.949 |
| AP-18 | 86.74±1.384 | 87.387±1.100 | 48.167±1.170 | 48.550±1.949 |
| AP-24 | 83.597±1.010 | 77.97±4.282 | 64.493±1.136 | 42.624±2.036 |
| AP-26 | 63.007±5.303 | 67.563±.818 | 47.487±0.527 | 38.777±0.788 |
| AP-27 | 85.577±1.720 | 72.553±.870 | 64.597±1.967 | 20.706±1.878 |
| AP-29 | 63.557±2.694 | 45.55±2.073 | 45.54±1.539 | 43.539±1.362 |
| AP-31 | 65.09±2.694 | 66.903±3.216 | 34.437±1.359 | 24.648±1.987 |
| AP-33 | 60.467±5.530 | 75.453±0.566 | 58.92±1.502 | 0.331±0.832 |
| AP-34 | 38.613±2.058 | 55.8±2.305 | 34.907±3.111 | 22.486±1.724 |
| AP-35 | 60.367±1.381 | 22.277±2.261 | 37.117±1.129 | -10.886±1.714 |
| AP-39 | 22.843±3.091 | 60.367±1.381 | 38.367±4.844 | 21.481±1.079 |
| AP-42 | 92.557±1.957 | 88.393±2.563 | 78.933±0.510 | 63.528±1.563 |
| AP-46 | 50.66±0.767 | 50.75±1.756 | 52.54±2.529 | 2.770±0.968 |
| AP-47 | 31.883±2.701 | 33.07±2.570 | 31.757±2.179 | 17.175±1.179 |
| AP-48 | 50.46±2.282 | 44.08±1.518 | 48.677±0.520 | 22.043±0.638 |
| AP-50 | 70.973±2.768 | 53.737±2.912 | 64.793±3.330 | 8.972±1.563 |
| AP-56 | 48.273±3.055 | 53.263±2.870 | 45.843±2.155 | -13.181±1.252 |
| AP-61 | 37.85±3.262 | 28.027±2.946 | 36.477±2.111 | -15.912±2.047 |
| AP-63 | 48.93±3.189 | 57.483±1.046 | 55.993±2.478 | 9.479±0.800 |
| AP-65 | 36.007±1.162 | 37.207±1.085 | 57.827±3.024 | 10.353±1.006 |
| AP-69 | 91.337±2.9744 | 86.273±2.120 | 93.72±1.239 | 13.505±1.891 |
| AP-76 | 27.957±2.571 | 15.553±2.611 | 43.41±1.113 | -5.282±0.468 |
| AP-81 | 89.117±1.229 | 89.313±1.959 | 86.68±1.790 | 51.602±2.809 |
| AP-89 | 42.777±2.694 | 65.497±1.211 | 64.013±1.665 | 46.704±2.084 |
| AP=104 | 84.627±0.717 | 43.52±3.028 | 38.94±0.768 | -19.527±2.143 |

# Supplementary Table 2 DPPH radical scavenging rate of different concentrations of EXEs (n=3).

| concentration  （mg/mL） | Y1 | Y4 | Y12 | Y42 | Y69 | V*c* |
| --- | --- | --- | --- | --- | --- | --- |
| 20.000 | 95.503±0.494 | 89.87±2.439 | 94.797±2.015 | 94.797±1.072 | 88.77±0.145 | 99.567±0.100 |
| 10.000 | 95.347±0.902 | 80.247±2.117 | 92.863±0.939 | 78.72±4.105 | 85.92±1.531 | 99.437±0.021 |
| 4.000 | 95.17±0.737 | 75.277±2.983 | 84.74±2.645 | 65.31±1.828 | 75.907±2.812 | 99.587±0.199 |
| 2.000 | 85.57±2.102 | 52.05±1.567 | 76.913±4.031 | 49.663±3.615 | 60.703±1.765 | 96.617±0.811 |
| 1.000 | 74.623±1.831 | 33.027±0.048 | 59.43±3.098 | 36.143±3.046 | 46.613±2.409 | 94.407±1.798 |
| 0.500 | 55.213±3.448 | 17.503±2.744 | 42.05±4.567 | 11.863±0.342 | 26.257±3.144 | 89.107±0.227 |
| 0.250 | 20.943±2.281 | 2.583±0.605 | 16.36±3.124 | 2.47±0.035 | 9.12±1.910 | 81.33±1.055 |
| 0.125 | 11.98±0.376 | 0.97±0.550 | 7.503±2.744 | 0.497±0.290 | 0.283±0.160 | 77.553±1.233 |

# Supplementary Table 3 ABTS radical scavenging rate of different concentrations of EXEs (n=3).

| concentration  （mg/mL） | Y1 | Y4 | Y12 | Y42 | Y69 | V*c* |
| --- | --- | --- | --- | --- | --- | --- |
| 20.000 | 95.647±0.969 | 95.247±1.085 | 87.387±1.100 | 88.393±2.563 | 89.313±1.959 | 99.42±0.157 |
| 10.000 | 86.54±1.530 | 93.357±1.051 | 76.767±2.162 | 82.91±2.623 | 72.3±1.482 | 99.413±0.196 |
| 4.000 | 64.17±1.625 | 87.657±1.131 | 67.01±1.795 | 70.727±2.034 | 53.837±3.004 | 99.387±0.055 |
| 2.000 | 59.04±3.547 | 76.017±1.684 | 56.063±2.353 | 56.96±2.078 | 47.087±1.073 | 99.387±0.146 |
| 1.000 | 45.647±2.744 | 63.46±1.707 | 41.05±3.139 | 49.293±0.879 | 25.933±1.703 | 95.617±0.562 |
| 0.500 | 33.83±1.490 | 54.197±1.561 | 18.497±1.996 | 33.313±3.085 | 13.413±1.938 | 89.03±0.208 |
| 0.250 | 19.187±1.460 | 35.017±0.581 | 9.103±0.641 | 17.803±2.420 | 3.803±0.545 | 85.16±1.814 |
| 0.125 | 6.817±2.245 | 21.55±2.073 | 1.883±0.588 | 4.787±0.589 | 0.613±0.571 | 76.58±0.816 |

# Supplementary Table 4 OH radical scavenging rate of different concentrations of EXEs (n=3).

| concentration  （mg/mL） | Y1 | Y4 | Y12 | Y42 | Y69 | V*c* |
| --- | --- | --- | --- | --- | --- | --- |
| 20.000 | 92.747±2.072 | 82.65±4.218 | 95.537±0.957 | 93.72±1.239 | 86.68±1.790 | 99.603±0.163 |
| 10.000 | 83.133±1.590 | 73.77±1.050 | 73.043±2.295 | 62.533±2.194 | 76.597±1.796 | 99.423±0.072 |
| 4.000 | 65.427±1.697 | 63.23±2.401 | 62.02±2.036 | 46.307±3.370 | 64.207±1.628 | 96.013±0.626 |
| 2.000 | 58.18±1.252 | 44.283±1.288 | 52.603±0.902 | 38.417±0.595 | 47.503±1.037 | 92.383±1.100 |
| 1.000 | 44.56±1.129 | 25.52±1.875 | 44.583±4.618 | 14.763±0.936 | 35.207±2.770 | 85.733±3.129 |
| 0.500 | 33.413±1.280 | 9.497±0.808 | 37.817±5.072 | 0.287±0.170 | 19.057±2.482 | 75.194±2.892 |
| 0.250 | 13.847±1.084 | 2.33±1.374 | 13.41±1.011 | 0.037±0.015 | 12.01±2.575 | 60.69±1.548 |
| 0.125 | 0.18±0.165 | 0.154±0.166 | 0.41±0.066 | 0.027±0.015 | 3.133±0.645 | 47.223±1.383 |

# Supplementary Table 5 PTIO radical scavenging rate of different concentrations of EXEs (n=3).

| concentration  （mg/mL） | Y1 | Y4 | Y12 | Y42 | Y69 | VC |
| --- | --- | --- | --- | --- | --- | --- |
| 20.000 | 67.857±1.805 | 46.127±3.114 | 60.397±2.041 | 63.997±2.195 | 51.967±2.036 | 99.317±0.271 |
| 10.000 | 48.323±1.511 | 17.55±5.844 | 37.61±0.992 | 56.377±2.095 | 33.427±2.728 | 99.157±0.064 |
| 4.000 | 22.877±1.814 | 0.417±0.055 | 8.867±1.339 | 35.903±3.079 | 14.12±0.642 | 93.657±1.510 |
| 2.000 | 13.77±1.082 | 0.533±0.152 | 1.033±0.758 | 8.28±1.266 | 13.143±2.348 | 85.357±1.330 |
| 1.000 | 5.343±1.828 | 0.05±0.017 | 0.167±0.185 | 1.56±0.200 | 0.59±0.296 | 76.193±1.181 |

# Supplementary Table 6 Diameter of inhibition zone of 32 endophytic fungi mycelial extraction (MEs) against *S. aureus*, *B. subtilis*, *E. coli*, and *P. aeruginosa* (n=3).

| Strains | Diameter of inhibition zone^*^ (mm) | | | |
| --- | --- | --- | --- | --- |
|  | *S. aureus* | *B. subtilis* | *E. coli* | *P.aeruginosa* |
| AP-1 | 4.115±0.583 | 19.67±2.077 | 2.269±1.252 | 3.610±0.804 |
| AP-3 | - | 9.496±0.837 | - | 2.282±1.69 |
| AP-4 | 25.472±2.613 | 19.021±1.822 | 17.472±0.956 | 11.789±1.279 |
| AP-8 | 12.295±1.983 | 9.338±2.363 | 1.505±1.142 | 3.910±1.128 |
| AP-10 | - | - | - | - |
| AP-11 | - | 12.419±2.141 | 0.615±0.32 | 7.925±0.527 |
| AP-12 | 27.023±1.551 | 14.771±1.694 | 12.488±1.231 | 12.285±0.465 |
| AP-15 | 15.043±1.240 | 3.752±0.478 | 1.457±0.973 | - |
| AP-18 | 15.384±2.699 | - | - | 2.451±0.484 |
| AP-24 | 17.637±1.236 | - | - | - |
| AP-26 | 2.064±0.398 | 6.183±1.059 | 1.805±1.106 | - |
| AP-27 | 16.599±1.880 | 14.885±1.363 | 12.104±1.553 | 2.726±1.126 |
| AP-29 | 15.874±2.263 | 12.012±1.714 | - | 21.832±0.545 |
| AP-31 | 10.994±1.521 | 21.926±2.347 | - | 7.654±1.300 |
| AP-33 | - | - | - | - |
| AP-34 | 9.242±0.743 | - | 12.663±0.942 | 1.499±1.957 |
| AP-35 | 8.528±1.884 | 10.485±1.677 | 11.065±1.382 | 12.717±0.699 |
| AP-39 | - | - | 6.248±1.288 | - |
| AP-42 | 6.316±1.571 | 17.823±1.177 | - | - |
| AP-46 |  | - | - | - |
| AP-47 | 35.266±2.987 | 15.719±1.121 | 12.663±0.942 | 29.570±2.569 |
| AP-48 | 32.533±1.197 | 6.826±1.089 | 11.968±1.171 | 21.190±1.904 |
| AP-50 | 23.489±1.105 | 10.957±1.320 | 8.089±1.238 | - |
| AP-56 | 24.734±2.830 | 9.060±1.480 | - | - |
| AP-61 | - | 14.652±2.086 | - | - |
| AP-63 | - | - | - | - |
| AP-65 | - | - | - | - |
| AP-65 | 9.093±2.999 | - | 1.555±0.855 | - |
| AP-69 | 20.512±2.035 | - | - | - |
| AP-76 | 24.407±2.027 | 1.553±1.079 | - | - |
| AP-81 | 19.543±1.185 | 14.722±2.513 | 13.892±1.501 | - |
| AP-89 | - | - | - | 9.736±2.720 |
| AP-104 | 19.565±0.742 | 31.504±0.893 | 15.899±0.424 | 22.392±0.964 |
| DAB | 4.115±0.583 | 19.67±2.077 | 2.269±1.252 | 3.610±0.804 |

***** The diameter of the inhibition zone is the value after subtracting the diameter of the Oxford cup (6mm).

Note: “-” means no antibacterial activity, “DAB” means positive control-double antibody.

# Supplementary Table 7 AP-4 and AP-12 HPLC retention time and peak area (n=3).

|  | Ingredients to be tested | Retention Time | | | Peak Area | | |
| --- | --- | --- | --- | --- | --- | --- | --- |
|  |  | 1 | 2 | 3 | 1 | 2 | 3 |
| Andrographolide Standard |  | 25.871 | 26.008 | 25.897 | 2890365 | 2772870 | 2828867 |
| AP | AD | 25.744 | 25.742 | 25.734 | 12339836 | 11927367 | 11827676 |
|  | NAD | 47.941 | 47.927 | 47.936 | 188033 | 187936 | 172836 |
|  | DAD | 54.19 | 54.204 | 54.216 | 3203741 | 3352417 | 3382764 |
|  | DDAD | 56.126 | 56.134 | 56.17 | 235269 | 263542 | 243564 |
| AP-4 first generation | AD | 25.78 | 25.764 | 25.803 | 5649725 | 5872634 | 5500974 |
|  | NAD | 47.919 | 48.032 | 48.124 | 1365281 | 1273659 | 1192860 |
|  | DAD | 54.192 | 55.725 | 55.763 | 345830 | 339874 | 326547 |
|  | DDAD | 56.131 | 56.236 | 57.095 | 228539 | 203865 | 187304 |
| AP-12 first generation | AD | 26.055 | 27.361 | 26.65 | 5500194 | 5263998 | 5426381 |
|  | NAD | 48.213 | 48.836 | 47.624 | 545023 | 537264 | 572836 |
|  | DAD | 54.517 | 54.367 | 54.253 | 348271 | 374652 | 336254 |
|  | DDAD | 56.465 | 56.367 | 56.893 | 226210 | 203875 | 186273 |
| AP-4 second generation | AD | 25.672 | 24.995 | 25.036 | 590663 | 532846 | 583946 |
|  | NAD | 46.978 | 47.642 | 47.065 | 87154 | 84638 | 86901 |
|  | DAD | 54.116 | 54.367 | 54.378 | 860546 | 836404 | 864723 |
|  | DDAD | - | - | - |  |  |  |
| AP-12 second generation | AD | 25.689 | 25.673 | 25.874 | 679890 | 623749 | 653748 |
|  | NAD | - | - | - |  |  |  |
|  | DAD | 54.138 | 54.273 | 54.985 | 423213 | 409374 | 382647 |
|  | DDAD | - | - | - |  |  |  |

Note: “-” means no data detected.

1. **Supplementary Figures**

**
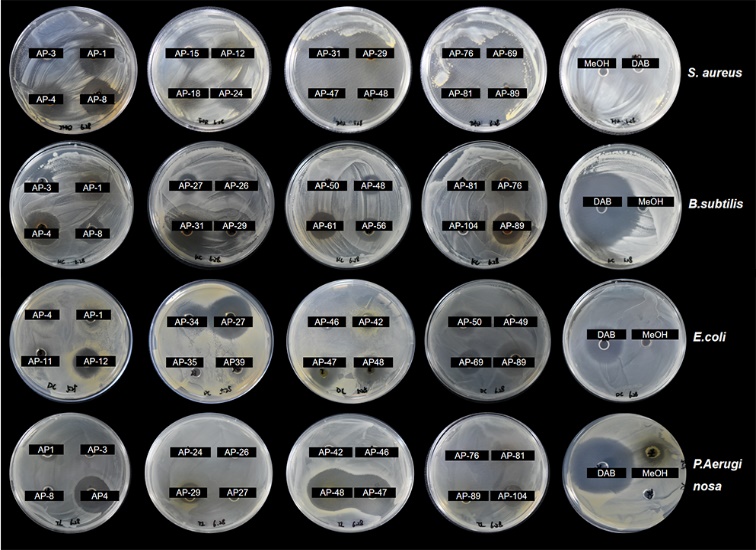
**

**Supplementary Figure 1** Oxford Cup bacteriostasis experiment of 32 dominant endophytic fungi mycelial extractions (MEs) against *E. coli*, *Pseudomonas aeruginosa*, *S. aureus*, and *Bacillus subtilis*.

**Supplementary Figure 2** MBC of AP-4, AP-12, AP-47, and AP-48 mycelial extractions (MEs) against E. coli, Pseudomonas aeruginosa, S. aureus, and Bacillus subtilis.
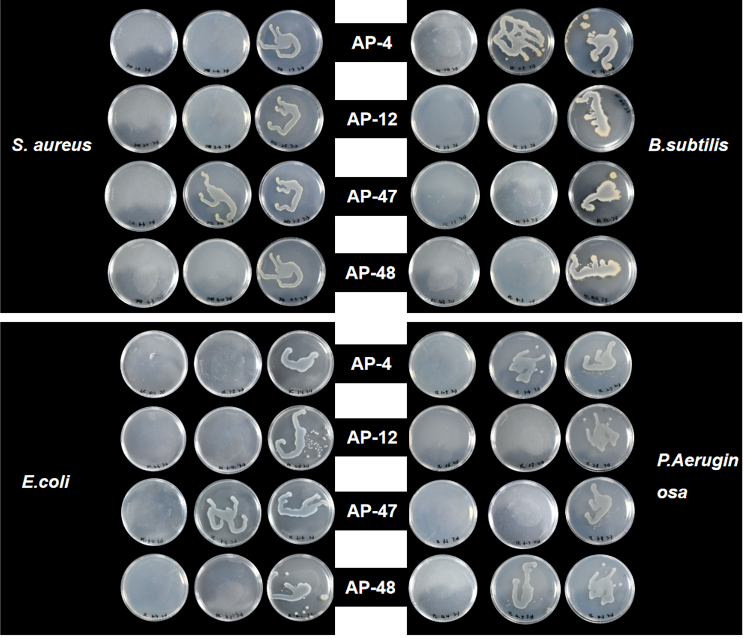

Supplement: Supplementary file 1 [file Data_Sheet_1.docx]
